# GLUT4 content decreases along with insulin resistance and high levels of inflammatory markers in rats with metabolic syndrome

**DOI:** 10.1186/1475-2840-11-100

**Published:** 2012-08-16

**Authors:** Natalia M Leguisamo, Alexandre M Lehnen, Ubiratan F Machado, Maristela M Okamoto, Melissa M Markoski, Graziela H Pinto, Beatriz D Schaan

**Affiliations:** 1 Laboratório de Experimentação Animal e Laboratório de Cardiologia Molecular e Celular, Instituto de Cardiologia/Fundação, Universitária de Cardiologia, Av. Princesa Isabel, 395. Bairro Santana, Porto Alegre, RS, 90620 001, Brazil; 2 Departamento de Fisiologia e Biofísica do Instituto de Ciências Biomédicas, Universidade de São Paulo, São Paulo, Brazil; 3 Serviço de Endocrinologia, Hospital de Clínicas de Porto Alegre, Universidade Federal do Rio Grande do Sul, Porto Alegre, Brazil

**Keywords:** Monosodium glutamate, Spontaneously hypertensive rats, Glucose transporter 4

## Abstract

**Background:**

Metabolic syndrome is characterized by insulin resistance, which is closely related to GLUT4 content in insulin-sensitive tissues. Thus, we evaluated the GLUT4 expression, insulin resistance and inflammation, characteristics of the metabolic syndrome, in an experimental model.

**Methods:**

Spontaneously hypertensive neonate rats (18/group) were treated with monosodium glutamate (MetS) during 9 days, and compared with Wistar-Kyoto (C) and saline-treated SHR (H). Blood pressure (BP) and lipid levels, C-reactive protein (CRP), interleukin 6 (IL-6), TNF-α and adiponectin were evaluated. GLUT4 protein was analysed in the heart, white adipose tissue and gastrocnemius. Studies were performed at 3 (3-mo), 6 (6-mo) and 9 (9-mo) months of age.

**Results:**

MetS rats were more insulin resistant (p<0.001, all ages) and had higher BP (3-mo: p<0.001, 6-mo: p = 0.001, 9-mo: p = 0.015) as compared to C. At 6 months, CRP, IL-6 and TNF-α were higher (p<0.001, all comparisons) in MetS rats *vs* H, but adiponectin was lower in MetS at 9 months (MetS: 32 ± 2, H: 42 ± 2, C: 45 ± 2 pg/mL; p<0.001). GLUT4 protein was reduced in MetS as compared to C rats at 3, 6 and 9-mo, respectively (Heart: 54%, 50% and 57%; Gastrocnemius: 37%, 56% and 50%; Adipose tissue: 69%, 61% and 69%).

**Conclusions:**

MSG-treated SHR presented all metabolic syndrome characteristics, as well as reduced GLUT4 content, which must play a key role in the impaired glycemic homeostasis of the metabolic syndrome.

## Background

Metabolic syndrome is a highly prevalent condition [1] and a determinant of increased cardiovascular risk [2] and type 2 diabetes [3]. Insulin resistance is the key factor that leads to several of the abnormalities associated with the syndrome [4]. The link between insulin resistance and metabolic syndrome was suggested to be inflammation [5], which is the most widely accepted hypothesis for its development [5-9]. Besides, hypertension is related to insulin resistance [4], a feature that can be genetically induced [10,11].

GLUT4 is the insulin-sensitive glucose transporter which main role is to provide the insulin-stimulated glucose uptake by adipose tissue, skeletal muscle and the heart, tissues that specifically express this protein [12]. It has been extensively reported that transgenic mice lacking or overexpressing GLUT4 respectively, decrease or increase whole-body insulin sensitivity [13], pointing out its role in the maintenance of glucose homeostasis. In obesity, the reduction of this transporter gene expression is directly related to the development of insulin resistance [14].

Inflammatory cytokines produced by the adipose tissue, such as TNF-α (tumor necrosis factor-α) and interleukin-6 (IL-6) have been related to reduce GLUT4 expression [8,9], consequently lowering glucose uptake by muscle, and participating on the compensatory hyperinsulinemia [7,15,16]. Administration of monosodium glutamate (MSG) to rats [17] or mice [18] is a well-known animal model of obesity. Obesity development in adult rodents induced by neonatal injection of MSG was first proposed by Nemeroff and cols. [19]. This treatment induces neuroendocrine dysfunctions as a consequence of lesion in the arcuate nucleus of the hypothalamus, which compromises dopaminergic and cholinergic tubero-infudibular systems [20,21]. The neurotoxic effect of MSG is restricted to the neonatal period, because of the immature blood–brain barrier [22], and seems to be dose-dependent [23,24]. Hypophagia is usually observed [24,25], and obesity derives from a lower metabolic rate [26], related to decreased thermogenesis [27], attributed to low sympathetic nervous system activity of brow adipose tissue [28]. Endocrine alterations such as decreased plasma growth hormone concentration [21] and increased plasma corticosterone concentration [23] can contribute to the development of obesity. Glucose homeostasis derangements seem to be more severe in mice than in rats; mice can develop diabetes [29,30], and rats an insulin-resistant state without hyperglycemia [31]. Since the description of this animal model in the late seventies, it has been extensively used to investigate the pathophysiology of obesity and its potential therapeutic approaches.

On the other hand, MSG treatment of spontaneously hypertensive rats (SHR) could approach a classical animal model of the metabolic syndrome, by association of obesity with arterial hypertension, but there are reports that high blood pressure (BP) levels are attenuated in these animals [32]. Moreover, this animal model was not fully characterized as to the development and maintenance of the metabolic syndrome features over time. We hypothesized that the induction of obesity in hypertensive rats would determine a cluster of dysfunctions enough to characterize the metabolic syndrome, as it is observed in humans, pointing out reduced expression of GLUT4 in insulin-sensitive tissues as a marker of insulin resistance. Thus, the aim of this study was to characterize the metabolic syndrome in MSG-treated spontaneously hypertensive rats (SHR), focusing on GLUT4 protein expression and insulin resistance development, as well as on inflammatory cytokines and BP levels over time.

## Methods

All animals were bred and kept under standard laboratory animal house conditions at the Animal Production and Research Unit of the Center for Scientific and Technological Development of Fundação Estadual de Produção e Pesquisa em Saúde do Rio Grande do Sul, Brazil. The study was approved by the Research Ethics Committee of Instituto de Cardiologia do RS, protocol #UP:4330. Animals received standard rat food and water *ad libitum*, and were maintained in controlled 12-h light/12-h dark cycle (6AM/6PM) and 20-25°C temperature conditions.

Neonate male SHR (n = 18) were submitted to subcutaneous administration of monosodium glutamate (MSG, Sigma®) diluted in saline solution (0.9% NaCl), 5mg/g/day, for 9 days (MetS), starting at day one of life. We also evaluated 18 SHR (group H) and 18 Wistar-Kyoto rats (group C) treated with saline solution subcutaneously for the same period. At 21 days of life, the animals were weaned and placed into plastic boxes, 4 animals per box.

General characteristics, insulin sensitivity (insulin tolerance test) and blood pressure (analyzed on a beat-to-beat basis) were evaluated at 3, 6 and 9 months. The rats were euthanized with ketamine (160 mg/kg body weight) and xylazine (10 mg/kg body weight). Tissues (heart, epididymal white adipose tissue and gastrocnemius muscle) were removed for GLUT4 analyses (Western blotting), and blood was collected for lipid profile, C reactive protein (CRP), interleukin 6 (IL-6), tumor necrosis factor-α (TNF-α) and adiponectin analysis at the end of each period of evaluation, 6 animals of each group/period.

### General characteristics evaluation

The animals were weighed and their naso-anal lengths were measured in dorsal decubitus on the day they were euthanized. The Lee Index was calculated according to the formula: (weight ^1/3^/naso anal length) [30].

### Insulin tolerance test

The insulin tolerance test was performed as previously described [31] using human insulin (Humulin, Eli Lilly, São Paulo, Brasil). After 3 h of food restriction, animals were anesthetized with ketamine (160 mg/kg body weight) and xylazine (10 mg/kg body weight), and 0.75U/kg body-weight of regular insulin was injected via the penile vein. Glycemia was measured by Accu-check strips system (Roche, Mannheim, Germany) before insulin injection and 4, 8, 12, 16 and 20 minutes after. The glucose decay constant rate (kITT) was calculated as described [31].

### Blood pressure recording

The animals were anesthetized with ketamine (160 mg/kg body weight) and xylazine (10 mg/kg body weight) to place a polyethylene catheter (PE-10) inside the femoral artery. The cannula was filled with saline solution and positioned inside the abdominal aorta, through the left femoral artery, to record BP. The next day the arterial cannula was connected to a pressure transducer, linked by a channel selector to the CODAS analog-digital board in a microcomputer. The data obtained were recorded. For each pulse wave the same program calculated values for peak (systole), valley (diastole) and period (between one peak and the next), generating a spreadsheet with these values, which was analyzed in a Microsoft Excel 2007 software. Twenty-minute basal recordings were obtained to evaluate systolic and diastolic BP and heart rate, in the conscious animal.

### Tissues harvesting and euthanasia

One day after the BP recording, the animals were anesthetized as described. Gastrocnemius muscle and epididymal white adipose tissue were removed, and then the animals were submitted to a thoracotomy and cardiac puncture to draw blood, and further heart harvesting, causing their death. Serum was separated by centrifugation and frozen at −80°C. Tissues were weighed and immediately frozen for further analysis.

### Western-blotting for GLUT4 protein

The tissue samples were homogenized according to Machado et al. [29]. The gastrocnemius, heart and white adipose tissue were homogenized in buffer (10m*M* Tris–HCl, 1 mM EDTA and 250 m *M* saccarose, pH 7.4), using a Polytron homogenizer (Marconi, Piracicaba, Brazil) at 20,000 rpm for 30 seconds. Gastrocnemius and heart were homogenized in the same buffer (1:6 weight: volume), and centrifuged at 1,000 g for 10 min. The supernatant was saved; the pellet was resuspended in 1/3 of the initial volume, and centrifuged again at 1,000 g for 10 min. The two supernatant solutions were mixed and submitted to centrifugation at 150,000 g for 75 min. The final pellet was resuspended in 1 ml of buffer as a total membrane fraction. The white adipose tissue was homogenized in the same buffer (1:4 weight:volume), and centrifuged at 2,000 g for 15 minutes. The fat globules were discarded, and the volume of the infranatant, a fat-free extract fraction, containing the microssomal and the plasma membrane proteins (corresponding to total membrane fraction), was saved for further analysis. All procedures were carried out at 4 °C.

GLUT4 expression was determined by Western Blot. Briefly, The total protein concentration of the samples was determined by the Bradford method [33]. Equal quantities of total proteins (50 μg) were solubilized in Laemmli buffer, electrophorezed in sodium dodecyl sulfate/polyacrylamide gel electrophoresis (SDS-PAGE – 10%), and transferred to the nitrocellulose membrane (GE Healthcare, Amersham Biosciences, UK). After transfer, the membranes were blocked with non fat dried milk, and subjected to immunodetection using anti-GLUT4 antibody (Chemicon, Billerica, CA, USA) at a dilution of 1:3,000, 37°C for 3 hours. The immunoblots were revealed and visualized by enhanced chemiluminescence using ECL kit (GE Healthcare), and analyzed by optical densitometry using Image Quant TL software (GE Healthcare, New York, USA). For white adipose tissue analysis, the membranes were reprobed with anti-β-actin antibody (Monoclonal anti-β-actin antibody AC-74, A2228, Sigma-Aldrich), and GLUT4 values were normalized by the respective β-actin value. For heart and gastrocnemius analysis, densitometric analysis of total protein in the lanes was performed, between 35 and 130 kDa of range (based on Page Ruler Prestained Protein Ladder®, Thermo Scientific, USA), in Ponceau stained membranes. These values were used to normalize the respective GLUT4 values [34]. The final results were expressed as arbitrary units (AU).

### Lipid profile and inflammatory markers

Total cholesterol, HDL-cholesterol and triglycerides concentrations were analyzed using commercial kits (*Labtest®*, Lagoa Santa, MG, Brazil).

Immunodetection of inflammatory markers and adiponectin were analyzed by the immunoenzymatic method (ELISA) according to the respective manufacturer’s instructions: CRP (USA; *Ebiosciences®*, San Diego, CA USA), TNF-α ( *Cellsciences®*, Canton, MA, USA), IL-6 ( *Cellsciences®*, Canton, MA, USA) and adiponectin ( *Chemicon®*, Billerica, MA). The reading was obtained by spectrophotometry ( *Spectramax®*, Molecular Devices Corporation, Sunnyvale, CA, USA) at 450nm and compared to a standard curve obtained with known concentrations of recombinant mediators. The minimum values of detection, intra-assay variability coefficients and inter-assay variability coefficients according to the manufacturer were, respectively: CRP: 2.5 pg/mL, <8% and <7%; TNF-α: 25 pg/mL, <10% and <12%; IL-6: 2 pg/mL; <10% and <12%; adiponectin: 15.6 pg/mL, <10% and <10%.

### Statistical analysis

The results are presented as mean ± standard deviation and compared by two-way analysis of variance (ANOVA), followed by the Bonferroni´s post-hoc test. The level of significance was 5% for all tests performed. All analyses were performed using the SPSS for Windows, version 17.0.

## Results

General characteristics of the animals are shown in Table 1. Animals in the MetS group weighed less than C and H (p<0.001 for both comparisons) at 6 and 9 months of age. The heart and gastrocnemius mass were reduced in the MetS animals (p<0.001 for both comparisons). However, the weight of epididymal white fat was greater in the MetS group *vs.* C and H (p<0.001 for both comparisons) at all ages studied, and consequently, the Lee index was also higher in all ages (p<0.001), revealing the obesity of MetS rats. MetS rats had progressively increased triglycerides with aging (6 mo: p<0.001 MetS *vs.* C and p = 0.002 MetS *vs.* H; 9 mo: p<0.001 for MetS *vs.* C and H), as well as reduced HDL-cholesterol (6 mo: p<0.001 MetS *vs.* C and p = 0.014 MetS *vs.* H; 9 mo: p = 0.004 MetS *vs.* C and p = 0.009 MetS *vs.* H), with a more marked difference among groups at 9 months of age. Interestingly, H animals with 9 months of age also had increased triglyceride levels when compared with C (p<0.001). Total cholesterol of all groups was higher at 6 and 9 months when compared to 3 months of age, however, there were no differences among groups within each age.

**Table 1:** General characteristics of the animals studied at 3, 6 and 9 months of age

	**3 months**			**6 months**			**9 months**		
	**C**	**H**	**MetS**	**C**	**H**	**MetS**	**C**	**H**	**MetS**
Body weight (g)	262 ± 29	278 ± 9	254 ± 18	349 ±13	377 ± 11	291 ± 26 ^a^	355 ± 16	363 ± 11	305 ± 6 ^a^
Lee index	0.22 ± 0.1	0.26 ± 0.1	0.33 ± 0.1 ^a^	0.23 ± 0.1	0.27 ± 0.1	0.33 ± 0.1 ^a^	0.26 ± 0.1	0.29 ± 0.1	0.32 ± 0,1 ^a^
Heart weight (g)	0.91 ± 0.21	0.95 ± 0.11	0.80 ± 0.10 ^a^	1.12 ± 0.25	1.22 ± 0.18	0.90 ± 0.13 ^a^	1.22 ± 0.21	1.26 ± 0.19	0.80 ± 0.12 ^a^
Gastrocnemius weight (g)	1.39 ± 0.23	1.41 ± 0.30	1.10 ± 0.21	1.47 ± 0.21	1.59 ± 0.19	1.28 ± 0.14	1.61 ± 0.22	1.70 ± 0.26	1.31 ± 0.18 ^a^
WAT weight (g)	0.80 ± 0.16	0.88 ± 0.18	1.20 ± 0.17 ^a^	1.09 ± 0.18	1.19 ± 0. 21	1.42 ± 0.22 ^a^	1.26 ± 0.21	1.29 ± 0.31	1.66 ± 0.36 ^a^
Total cholesterol (mg/dl)	57 ± 12	56 ± 7	56 ± 7	70 ± 21	78 ± 21	76 ± 13	83 ± 7	77 ± 21	76 ± 13
HDL-c (mg/dl)	54 ± 4	54 ± 2	54 ± 2	55 ± 7	51 ± 3	37 ± 6 ^a^	53 ± 9	50 ± 7	39 ± 5 ^a^
Triglycerides (mg/dl)	49 ± 13	49 ± 15	49 ± 15	56 ± 6	58 ± 18	104 ± 19 ^a^	66 ± 11	170 ± 41 ^a^	444 ± 64 ^a^

*C*: Wistar-Kyoto rats that did not receive any treatment; *H*: spontaneously hypertensive rats that did not receive any treatment; *MetS*: spontaneously hypertensive rats that received MSG during the neonatal period. *WAT*: white adipose tissue; *HDL-c*: high density lipoprotein cholesterol. The table shows only differences among groups at the same time of evaluation, not inside groups throughout time. Differences among groups over time are described in the results section. ^a^ p<0.05 *vs.* C. Two-way ANOVA followed by Bonferroni’s post hoc test.

**Figure 1:**
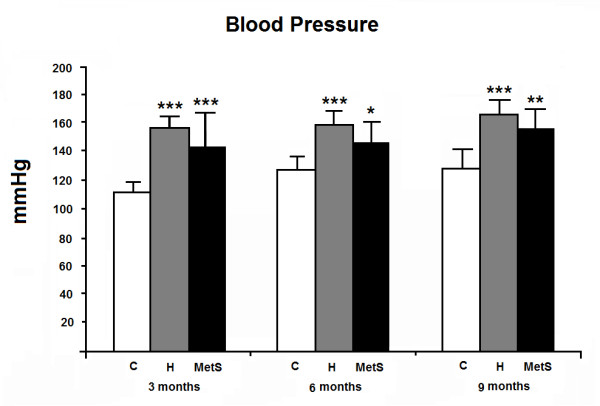
**Mean blood pressure of the animals studied, at 3, 6 and 9 months of age.** C: Wistar-Kyoto rats that did not receive any treatment; H: spontaneously hypertensive rats that did not receive any treatment; MetS: spontaneously hypertensive rats that received MSG during the neonatal period. n = 6 in all groups. Two-way analysis of variance (ANOVA): group (p<0.001), time (p = 0.431) and interaction (p = 0.016), followed by the Bonferroni’s post hoc test: * p<0.05, ** p<0.01 and *** p<0.001 *vs.* C at the same time.

Mean BP was similarly higher in H and MetS groups at all ages, as compared to the C group (Figure 1). There were no changes in BP over time in the groups studied.

**Figure 2:**
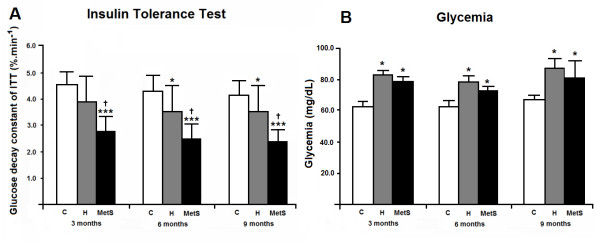
**Insulin sensitivity and glycemia of the animals studied, at 3, 6 and 9 months of age.** C: Wistar-Kyoto rats that did not receive any treatment; H: spontaneously hypertensive rats that did not receive any treatment; MetS: spontaneously hypertensive rats that received MSG during the neonatal period. n = 6 in all groups. Painel **A**: Insulin sensitivity evaluated by the insulin tolerance test (ITT). Two-way analysis of variance (ANOVA): group (p<0.001), time (p = 0.062) and interaction (p = 0.022), followed by the Bonferroni’s post hoc test: * p<0.05 and *** p<0.001 *vs.* C; † p<0.05 *vs.* H at the same time. Painel **B**: Glycemia after 8 hours of food deprivation. Two-way analysis of variance (ANOVA): group (p = 0.001), time (p = 0.021) and interaction (p = 0.041), followed by the Bonferroni’s post hoc test: * p<0.05 *vs.* C at the same time.

Figure 2 shows the results of the insulin tolerance test (Panel A) and glycemia (Panel B) after 8 hours of food deprivation. Animals from the MetS group were insulin-resistant at all ages, as compared to respective C and H groups although insulin resistance did not increase over time. Furthermore, H animals become insulin-resistant at 6 and 9 months of age, as compared to C (no difference between C and H was observed at 3 months of age). Glycemia was higher in H and MetS groups at all ages as compared to the C group (Figure 2, Panel B).

**Figure 3:**
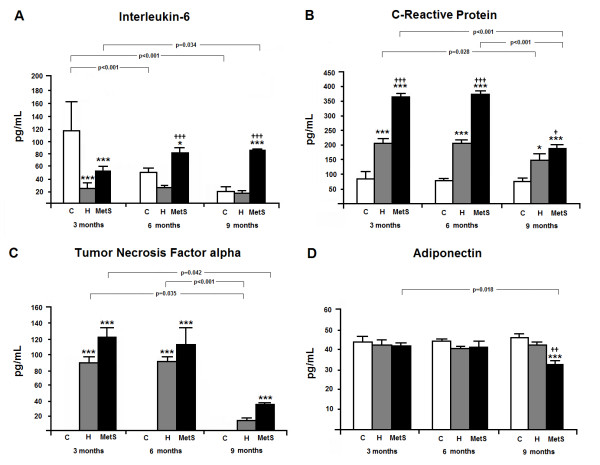
**Inflammatory markers of the animals studied, at 3, 6 and 9 months of age.** C: Wistar-Kyoto rats that did not receive any treatment; H: spontaneously hypertensive rats that did not receive any treatment; MetS: spontaneously hypertensive rats that received MSG during the neonatal period. n = 5 in all groups. Two-way analysis of variance (ANOVA) Panel **A**: group (p<0.001), time (p = 0.001) and interaction (p<0.001); Panel **B**: group, time and interaction (p<0.001); Panel **C**: group (p<0.001), time (p<0.001) and interaction (p = 0.035) and Panel ** D**: group, time and interaction (p<0.001), followed by the Bonferroni’s post hoc test: * p<0.05 and *** p<0.001 *vs.* C; †† p<0.01 and ††† p<0.001 *vs.* H at same time. The time course changes inside groups are also showed.

Figure 3 shows the results of the cytokines (TNF-alpha and IL-6), as well as CRP and adiponectin. IL-6 (Panel A) was elevated in MetS animals at 6 and 9 months of age, as compared to both C and H animals. A curious result was observed at 3 month of age, when IL-6 was lower in MetS and H animals, as compared do C. CRP (Panel B) and TNF-alpha (Panel C) were also higher in MetS animals in all ages, as compared do C and H animals; indeed, these cytokines were elevated in H, as compared to C at all ages, except at 9 months for TNF-alpha. Finally, adiponectin (Panel D) was altered only at 9 months of age, when it was reduced in MetS animals, as compared to both C and H animals.

**Figure 4:**
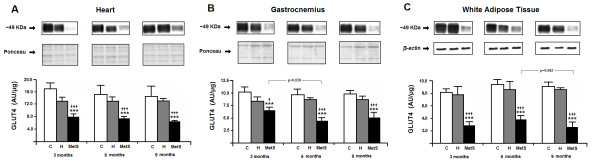
**Total GLUT4 protein in heart (A), gastrocnemius muscle (B) and epididymal white adipose tissue (C) from the animals studied.** C: Wistar-Kyoto rats that did not receive any treatment; H: spontaneously hypertensive rats that did not receive any treatment; MetS: spontaneously hypertensive rats that received MSG during the neonatal period. On the top, representative images of GLUT4 and respective loading controls. Loading controls are β-actin for white adipose tissue, and total proteins of 35 to 130 kDa range of the Ponceau stained membrane for heart and gastrocnemius, as described in Methods. On the bottom, the graphs show means ± SEM of n = 5 animals. Two-way analysis of variance (ANOVA) Panel **A**: group, time and interaction (p<0.001); Panel **B**: group (p = 0.006), time (p<0.001) and interaction (p = 0.003); Panel **C**: group, time and interaction (p<0.001), followed by the Bonferroni’s post hoc test: *** p<0.001 *vs.* C; † p<0.05 and ††† p<0.001 *vs.* H at same time. The time course changes inside groups are also showed.

GLUT4 expression (Figure 4) was lower in heart (panel A), gastrocnemius (Panel B) and white adipose tissue (Panel C) at all ages in the MetS group, as compared to C and H. The reduction of GLUT4 in the heart of MetS rats was 54%, 50% and 57% at 3, 6 and 9 months of age, respectively, as compared to C group. In the gastrocnemius of Mets animals, GLUT4 was lower 37%, 56% and 50% as compared to C group at 3, 6 and 9 months, respectively. Finally, the GLUT4 in the adipose tissue MetS group showed a reduction of 69%, 61% and 69% at 3, 6 and 9 months of age, respectively.

## Discussion

This study showed a reduction of GLUT4 protein content of insulin-sensitive tissues in an animal model of metabolic syndrome, a fundamental mechanism to impair glucose uptake and glucose homeostasis. This regulation, not yet seen, began at 3 months of age, and was not reverted as time passed. Furthermore, this glucose transporter modulation was accompanied by inflammation, insulin resistance and hypertension, also here described in the same animal model.

The use of MSG in genetically hypertensive rats led these animals to progressively increase body adiposity and hypertriglyceridemia; besides, developing and maintaining insulin resistance, low HDL-cholesterol, high blood pressure levels, and inflammation throughout the period studied. Other animal models used showed that high-fat-fed SHRs did not show changes in plasma concentrations of total cholesterol and triglycerides, although plasma concentrations of free fatty acids were higher as compared to normal diet-fed SHRs [35]. Mice fed on high-carbohydrate and high-fat diet (cafeteria diet) had increased body weight, abdominal fat deposition, hyperinsulinaemia, hyperglycaemia and hyperleptinaemia, but no hypertension [36,37]. Although these are examples of models of metabolic syndrome [38], our study clearly shows the possibility of reproducing the whole metabolic syndrome in laboratory animals in a low-cost and easily-obtained model.

The increased Lee index and epididymal fat mass observed in MetS animals confirmed their obese condition, as it has been described in MSG-treated normotensive rats and mice [32,39]. MSG-treated animals, compared to their controls, may have a lower absolute weight, as we also found in MetS, which has been proposed to be a result of decreasing growth hormone (GH) secretion [40]. Accordingly, lean mass may be decreased in MSG animals [41], a feature that we also observed in SHR treated with MSG, which presented lower heart and skeletal muscle mass. In addition to low GH levels, high corticosterone levels [42] were also described in MSG animals, and both hormonal modulations contribute to decrease synthesis and increase catabolism of proteins, thus diminishing muscle mass [43]*.* Furthermore, together with decreasing sympathetic activity [28] this hormonal imbalance contributes to conserve energy, accumulating fat. It is important to highlight that MSG should be administered in the neonatal period to determine all the derangements related to obesity described, as use in later periods of life [44] would not result in the characteristic hypothalamic lesions [20,21], because of the protective effects of the blood–brain barrier [22].

In metabolic syndrome, however, beyond obesity, hypertension is a key feature, which is not present when MSG is used in mice [30] and Wistar rats [45], but was effectively shown in the present study treating SHR. Other authors described attenuation of high BP levels in MSG-treated SHR [32,39], a finding that we did not observe, probably because the cardiovascular evaluation method we used is more appropriate (direct BP measurement). The studies mentioned found controversial results, possibly due to the fact that all measured mean BP using tail plethysmography. Furthermore, variable protocols of neonatal MSG treatment have been used, probably altering severity and/or time of induction of the alterations. It is well-known that, in SHR, BP rises progressively over lifetime, while in our study, the MSG-treated SHR had higher BP than the normotensive rats at all ages studied, but remained stable, not increasing over time. This fact might be related to low sympathetic activity described in MSG-treated animals [28].

Fulfilling the picture of metabolic syndrome, MSG-treated SHR presented obesity and high BP levels associated with insulin resistance, hypertriglyceridemia and reduced HDL cholesterol levels with normal total cholesterol levels. Spontaneously hypertensive rats are typically insulin resistant [12], which we also observed in the MSG-treated SHR. However, SHR are not obese, and their visceral fat content is similar to that of normotensive Wistar rats [46], which was strongly changed by using MSG. Insulin resistance in SHR is partially ascribed to the characteristic sympathetic hyperactivity they present, which promotes reduced activity of the insulin signaling cascade and, consequently, can reduce GLUT4 translocation and/or expression [47]. Furthermore, norepinephrine inhibits insulin-mediated glucose uptake in muscle [48] and blocks insulin inhibitory action on liver glucose production [49], all these effects contributing to impair glycemic homeostasis. The characteristic lipid profile of insulin-resistant states was observed from 6 months of age and beyond for the MSG-treated SHR, as observed for non-MSG treated SHR [50], but not for Wistar-Kyoto rats. Based on these facts, we can understand that increased sympathetic activity may induce or worsen installed insulin resistance, closing the circle which perpetuates the existence of both insulin resistance and high BP levels.

Beyond its classic metabolic actions, insulin is also anti-inflammatory, decreasing activity of pro-inflammatory cytokines, such as TNF-α and IL-6, as well as repressing the transcription factor nuclear factor кB (NFкB) [51]*.* In MSG-treated mice, Furuya and collaborators [52] demonstrated that hypertrophyc adipocytes triggered local inflammatory activity with increased macrophage infiltration and TNF-α and IL-6 expression, depicting high plasma concentration of the cytokines. This was accompanied by decreased GLUT4 content in white adipose tissue and reversed by atorvastatin treatment [52]. We showed that inflammation is exacerbated in MSG-treated SHR from 3 months of age on, along with GLUT4 reduction in all insulin-sensitive tissues. TNF-α rise, especially, can reduce the expression of IRS-1 and GLUT4, as well as of the hormone-sensitive lipase, adiponectin and PPARγ [53]. All these processes are known to contribute to lipolysis and insulin resistance, and were also observed in these animals since the age of 3 months. Adiponectin reduction in MSG-treated SHR at the age of 9 months is probably due to the exacerbation of hypertriglyceridemia at this age, which acts as an independent causal factor for hypoadiponectinemia [54]. Besides, hypoadiponectinemia probably occurs only at the age of 9 months because of its known relationship with the severity of obesity [55].

Insulin resistance of MSG-treated SHR is in accordance with the reduction of GLUT4 content in all insulin-sensitive tissues analyzed, corroborating previous data in MSG-treated mice in all cell fractions of adipose tissue with no change in the relative GLUT4 translocation to the plasma membrane [30] and also in the same tissue in humans [56]. Moreover, low GLUT4 protein content in skeletal muscle and heart was also shown in MSG-treated mice [18]. In MSG-treated SHR, GLUT4 on insulin-sensitive tissues had not been reported yet, especially its time-course changes through aging, as here reported.

Skeletal and heart GLUT4 protein were reduced from the age of 3 months and did not worsen over time, following the stable profile of insulin resistance in MSG-treated SHR. It is known that in the SHR, plasma membrane myocyte GLUT4 increases with age (12 weeks), but at 20 weeks, GLUT4 contents tends to reach the initial levels (8 weeks) [57]. Moreover, reduced insulin-induced GLUT4 translocation [47] and/or total content was also showed before [57]. Pharmacological treatment of arterial hypertension with captopril can virtually re-establish the glucose transporter contents [57]. On the other hand, investigations have shown that GLUT4 translocation in skeletal muscle and heart is stimulated by bradykinin [58], which enhances insulin-induced phosphorylation of insulin receptors and insulin-stimulated association of IRS-1 and phosphatidylinositol-3-kinase in skeletal muscle of aged rats [59], all of which are essential for insulin-mediated GLUT4 translocation and glucose transport. Both cases demonstrate that the amount of GLUT4 is related to blood pressure levels. Anyhow, it is known that the behavior of this transporter in the skeletal muscle does not always reflect what happens in the white adipose tissue [60], and, indeed, in this tissue, we find a progressive reduction of its content in MSG-treated SHR, especially at the age of 9 months.

In the heart, in turn, it is known that insulin resistance together with the excess of free fatty acids – as we find in MSG-treated SHR – are responsible for contractility dysfunction [61,62]. Since the muscle contraction also induces GLUT4 translocation to the plasma membrane, contractility dysfunction is directly related to reduced plasma membrane GLUT4 content [63]. Furthermore, the excess of free fatty acids alone can interfere in glucose transport, since it reduces GLUT4 expression in the heart, but not that of the free fatty acid transporter [64]. In rats with insulin resistance induced by a fructose-rich diet, cardiomyocytes present low glucose input in response to ischemia, a consequence of GLUT4 translocation reduction [65]. We found a reduction of GLUT4 content in heart of MSG-treated SHR at the age of 3, 6 and 9 months, which can be explained by the hypothesis that the reduction of this transporter in the heart occurs when obesity and insulin resistance are established [41]. The normotensive and hypertensive control groups did not present differences in the GLUT4 content in the heart, at the age of 6 months [12]. In the MSG-treated SHR, time-course of changes of insulin-sensitive GLUT4 protein content had not yet been described in the literature.

## Conclusions

The present study depicts in obese hypertensive rats a reduction in GLUT4 expression, accompanied by whole-body insulin resistance, and increased plasma concentration of inflammatory markers. These findings characterize an animal model of metabolic syndrome, as it has been observed in humans. In general, the alterations have persisted unaltered during the aging process, from 3 to 9 months of age. Thus, the MSG-treated SHR can be used as an experimental model to investigate pharmacological approaches for the metabolic syndrome, as well as its interaction with other diseases.

## Competing interests

The authors declare that they have no competing interests.

## Authors’ contributions

NMM was involved in conception and design of the study, data collection, data analysis and interpretation, as well as drafting and editing the final document for publication. AML was involved in data analysis and interpretation, as well as drafting and editing the final document for publication. UFM was involved in conception and design of the study, data analysis and interpretation, as well as reviewing all parts of the final document for publication. GHP was involved in data collection, data analysis and interpretation. OMM and MMM were involved in data collection (molecular analysis), data analysis and interpretation. BDS was involved in conception and design of the study, data analysis and interpretation, as well as writing, drafting and editing the final document for publication. All authors read and approved the final manuscript.
